# Transfer learning for improving generalizability in predicting soybean maturity date using UAV imagery

**DOI:** 10.3389/fpls.2025.1720819

**Published:** 2026-01-29

**Authors:** Jing Zhou, Jianfeng Zhou, Andrew Scaboo, Eduardo Beche, Ziteng Xu, Zhou Zhang

**Affiliations:** 1 Department of Crop and Soil Science, Oregon State University, Corvallis, OR, United States; 2 Division of Plant Science and Technology, University of Missouri, Columbia, MO, United States; 3 College of Agriculture and Life Sciences, Texas A&M University, College Station, TX, United States; 4 Biological Systems Engineering, University of Wisconsin-Madison, Madison, WI, United States

**Keywords:** domain adaptation, maturity date, model generalizability, soybean breeding, transfer learning

## Abstract

**Introduction:**

High-throughput and accurate phenotyping is critical for enhancing crop breeding efficiency by enabling rapid identification of superior cultivars within large populations. For soybean [*Glycine max (L.) Merr.*], maturity group is a key determinant of geographic adaptation and influences yield potential. Consequently, accurate assessment of physiological maturity dates is essential for selecting lines suited to specific environments. This study evaluated the feasibility of three transfer learning techniques in improving the generalizability of models developed using historical data to predict the maturity dates of soybean breeding lines across new environments.

**Methods:**

Our dataset included five breeding trials conducted in two sites from 2018 to 2021. Maturity dates were visually assessed at the R8 stage, and multispectral imagery from an unmanned aerial vehicle (UAV) was collected within each trial. Seven image features served as predictors in the models. Transfer learning techniques, namely pre-training and fine-tuning, single-source and multiple-source domain adaptation, were evaluated using the multiple-year datasets.

**Results:**

When models were trained on data from three prior years and tested on two independent trials, the pre-training and fine-tuning technique demonstrated the best performance, with the highest agreement with visual ratings (coefficient of determination *R^2^* = 0.74 and 0.79) and root mean square errors of 1.70 and 1.96 days, respectively. The quantity for fine-tuning samples had minimal influence on the prediction accuracy for previously unseen data.

**Discussion:**

These findings provide a reference for leveraging accumulated knowledge to generalize deep learning models for future practical utilization.

## 1 Introduction

Soybean [*Glycine max (L.) Merr*.] represents one of the most essential crops to the world’s economy and food security due to its unique seed composition and versatile uses (Guo et al., 2022). Soybean meal, including protein, fiber, carbohydrates, and minerals, is intricately connected to the food supply through human food consumption and animal feed production, while soy oil provides great versatility with uses in food and beverage, wax, construction, cosmetics, plastics, and fuel (Shea et al., 2020). About 395 and 421 million metric tons of soybeans were produced worldwide in 2023 and 2024 production years, respectively (USDA Foreign Agricultural Service, 2025). However, due to large-scale world population growth combined with adverse agricultural environments, the demand for quality raw materials like soybean has increased, requiring highly productive cultivars and well environmental adaptation (Li et al., 2020; Santana et al., 2022).

Modern soybean breeding programs have been seeking elite soybean cultivars to ensure continued genetic gains in soybean grain yield as well as improve genetic diversity (Beche et al., 2020; Sun, 2014). Following specific selection criteria, elite cultivars are developed and selected from numerous crosses of modern cultivars as well as more diverse material including landraces and *G. soja* accessions with desirable agronomic characteristics (Fehr, 1991). Besides the primary trait (i.e., grain yield), physiological maturity date is another critical trait for selecting elite soybean cultivars. Physiological maturity concludes the reproductive phase, where seed number is set during the R1 to R4 stages of flowering and podding, and seed mass is set during the R5 to R6 stages of seed-filling (Board and Kahlon, 2011). As crop yield is reliant on the sum of photosynthetically active radiation absorbed by the crop over the course of a growing season and subsequently converted into harvestable grain yield, late-season radiation drops curtail the effective filling period, reducing harvest index and grain size (Rattalino-Edreira et al., 2020; Vogel et al., 2021). In addition, soybean production with delayed harvest is vulnerable to frost damage and poor seed quality, leading to harvest losses (Narayanan et al., 2019; Zhou et al., 2019). Therefore, soybean breeders categorize soybean varieties into maturity groups (MGs) to ensure that the cycle length of their selections aligns with the photoperiod and temperature conditions of the target region (Narayanan et al., 2019).

Soybean cultivars are divided into 10 MGs according to their time length from planting to physiological maturity or the R8 date, when 95% of their pods have reached mature color (Fehr, 1991). A concept of relative maturity is used to determine the MG of a new variety by referring its relative maturity to the commercially released soybean cultivars (referred to as checks) with a known MG (Mourtzinis and Conley, 2017). For example, relative maturity 3.0 - 3.9 are 10 subgroups in the MG III and the cultivars with relative maturity 3.0 mature earlier than the relative maturity 3.9 cultivars when planted at the same time and in the same environment. Relative maturity is determined by calculating the differences in the maturity dates (the first day of the year when soybean reaches R8) between the new cultivar and the relative maturity checks (Zhou et al., 2019). The dates of reaching R8 have been conventionally determined by breeders visually observing the colors of the pods, which is labor-intensive and time-consuming when facing numerous breeding materials across many environments.

The exploration of monitoring soybean maturity status for breeding using remote sensing and machine learning (ML) tools started in 2016. Yu et al. (2016) collected visible and near-infrared information on soybean breeding materials using an unmanned aerial vehicle (UAV)-based high-throughput phenotyping platform. A popular ML model, Random Forest (RF), was leveraged to distinguish between mature and immature soybean lines at the moment of data collection and finally achieved over 93% classification accuracy. Although with high accuracy, this dichotomy (i.e., determining whether a plot matured) approach was limited in practice as it requires frequent UAV field scouting with the appropriate timing for the scouting undetermined (Pérez et al., 2024). Inspired by Yu et al. (2016), our previous work in Zhou et al. (2019) presented the first approach capable of predicting the R8 date of soybean breeding lines from UAV multispectral imagery collected before R8. Our dataset consisted multiple times of UAV data collection with more than 100 multispectral image features as well as visually measured maturity dates of 326 soybean breeding lines. Leveraging feature engineering methods, significant image features for predicting maturity dates were determined as well as the best UAV data collection timing. Our best accuracy - coefficient of determination (*R^2^*) = 0.81 with a root mean square error *(RMSE)* of 1.4 days was given using 20 image features collected before and within the maturity stages. Our approach provided sufficient accuracy in determining soybean relative maturity with flexible UAV data collection timing in practices.

Following our work, several studies have explored alternative methods for determining soybean maturity, as summarized by Pérez (2025). These studies predominantly utilized UAV-derived RGB or multispectral imagery of soybean canopy, extracting features such as vegetation indices (VIs) and spectral band reflectance values. ML models, including partial least squares regression, random forest (RF), and convolutional neural networks (CNNs), were applied to predict maturity dates or classify binary maturity status based on image features captured on a specific date or time-series image sets. Among studies predicting precise maturity dates for soybean lines, the best performance was reported by Moeinizade et al. (2022), achieving an *R²* of 0.95 and *RMSE* of 0.9 days using data from six different environments.

Although several studies have predicted soybean maturity, only a few have assessed the generalizability of maturity prediction models by training on data from specific environments and testing on others. Trevisan et al. (2020) tested CNN models reciprocally using check cultivars to correct the bias in the raw predictions. Using RF instead, Pérez et al. (2024) tested the models on completely unseen data but also included the check cultivars from an independent environment in the cross-validation process, which limited the “unseen” evaluation. They concluded that the reliability of fitted models for predicting soybean physiological maturity in new environments hinges primarily on the similarity between training and testing conditions, with predictions in independent environments proving less accurate than those on subsets of the same environment.

For all crop characterization tasks adopting sensing and modeling, it is always the goal in methodological development that variations in crop traits caused by genotypic, environmental, geospatial, and even random variances can be tolerated or accounted for to deliver accurate estimates. Transfer learning has been proven to be highly effective in various areas, such as computer vision and natural language processing, by significantly enhancing model generalizability. In transfer learning, a model trained on one task or dataset can be repurposed or fine-tuned for a different but related task or dataset (Iman et al., 2023; Zhuang et al., 2021). The idea is to leverage the knowledge learned from one domain (e.g., crop data collected this year at one particular location) to improve the performance of a model on a different, yet related, domain (crop data collected the following years at the same or different locations).

Among various transfer learning techniques, pre-training and fine-tuning has been adopted in many applications due to its easy implementation and performance. This approach involves pre-training a model on a large dataset and then fine-tuning it on the target task. Popular models like BERT (Bidirectional Encoder Representations from Transformers) by Devlin et al. (2018) at Google and ChatGPT (Generative Pre-trained Transformer) by OpenAI (2023) are pre-trained on massive text corpora and then fine-tuned for specific downstream tasks. The technique can be applied to estimate soybean maturity date in future experiments by fine-tuning the models pre-trained on data collected in previous experiments. For receiving desired performance on estimates of future experiments, fine-tuning requires a certain amount of labeled training data, which will be available for determining soybean maturity because a group of checks with known relative maturity will always be planted in the experiments. Opposite to the pre-training and fine-tuning focusing on transferring the similarities, domain adaptation (DA) techniques were then introduced to address the difference (i.e., domain shift) between datasets or tasks among different domains (Ganin and Lempitsky, 2015). The DA technique is a specialized form of transfer learning (Zhao et al., 2022) that aims to learn a model from a labeled source domain (e.g., crop data collected in previous experiments) that can generalize well to a different (but related) unlabeled or sparsely labeled target domain (e.g., crop data collected in future experiments).

In the area of crop characterization for precision agriculture or high-throughput phenotyping, the pre-training and fine-tuning technique has been investigated in agricultural environment monitoring (Wang et al., 2023), diseases (Sanida et al., 2023; Yu et al., 2023), pests (Talukder et al., 2023), weed identification (Zhang et al., 2023), and leaf chlorophyll estimation (Yue et al., 2023). However, these implementations mainly intended to reduce the training time and improve accuracy in single domains, as these models were trained on benchmark datasets (Sanida et al., 2023) or simulated dataset (Yue et al., 2023). The potential of DA has been validated only for crop yield estimation at county and state scales (Liao et al., 2023; Ma et al., 2021; Priyatikanto et al., 2023) and for object detection tasks to mitigate the shifts caused by ambient light variations during imaging (Bertoglio et al., 2023; Chen et al., 2023; Fujisawa et al., 2023; Magistri et al., 2023; Zhao et al., 2023). No studies have been conducted adopting the DA for crop characterization tasks involving large genotypic, environmental, geospatial variations and their interactions.

Therefore, this study aimed to improve the generalizability of ML models in predicting soybean maturity date for unseen datasets by exploring transfer learning techniques. To reach the overall goal, we (1) developed multi-year datasets with UAV imagery and ground measurements for soybean maturity date prediction, (2) applied three transfer learning techniques and compared their prediction accuracy, and (3) evaluated the effects of key parameters in the transfer learning techniques on their generalizability, providing a reference for future practical utilization.

## 2 Materials and methods

### 2.1 Plant material

This study utilized soybean materials from five trials conducted at University of Missouri (MU) research facilities in Missouri, USA. The trials included a progeny row trial (PT) planted on May 29, 2018 at the Greenley Research Center (Novelty, MO); a preliminary yield trial (PYT) planted on June 3, 2019 and an advanced yield trial (AYT1) planted on June 2, 2020, both at the Bay Farm Research Facility (Columbia, MO); and two additional advanced yield trials, AYT2 and AYT3, planted on June 6 and 15, 2021, respectively, at the Greenley Research Center. The PT comprised 325 breeding lines and 108 check plots arranged in single rows. The PYT and AYT trials were planted in four-row plots, with PYT including 1,103 breeding lines and 162 checks, AYT1 with 778 breeding lines and 107 checks, AYT2 with 2,628 breeding lines and 396 checks, and AYT3 with 2,260 breeding lines and 124 checks. No breeding lines overlapped between AYT2 and AYT3 or between those ones and those from 2018–2020. A total of 7901 samples were used to construct the dataset.

Field management followed standard soybean breeding protocols in University of Missouri. Crop rows were planted 2.59 m in length in PT and 3.66 m in length in PYT and AYTs. Row spacing in all trials was 0.76 m. Seeding rate was 30 seeds per meter for all trials. The plots were harvested using a plot combine during late October to early November each year. All fields were prepared under conventional tillage with pesticide applications and irrigation as needed. All experimental fields located in a humid subtropical climate region (Köppen climate classification code: Cfa). Average temperature during planting dates was between 18.2-29.2°C with a monthly average precipitation of 107 mm.

### 2.2 UAV image collection and image features

Aerial imagery was captured using an imaging system featuring a UAV platform (model: DJI Matrice 600 Pro, DJI, Shenzhen, Guangdong, China) equipped with a multispectral camera (RedEdge-M, MicaSense, Seattle, WA, USA). The multispectral camera records five spectral bands (blue, green, red, rededge, near-infrared) at an image resolution of 1280×960 pixels. The camera was set to take images at one frame per second. Calibration was performed using a calibration reflectance panel (CRP, Model: RP04, Micasense, Seattle, WA, USA) following an established protocol (Zhou et al., 2019). The camera has an integrated Global Navigation Satellite System (GNSS) receiver that automatically embedded geo-referencing data into each image’s metadata. Prior to each mission, the calibration panel was imaged from approximately 1 m above in an open area to ensure shadow-free conditions.

Images were collected during late growth stages across the trials to evaluate physiological maturity. From 2018 to 2020, collections occurred twice per year: for PT on September 14 (108 days after planting or DAP) and 27 (121 DAP); for PYT on September 20 (109 DAP) and October 1 (120 DAP); and for AYT1 on September 17 (107 DAP) and 30 (120 DAP). In 2021, data from AYT2 and AYT3 were collected once on September 23, corresponding to 109 DAP and 100 DAP, respectively.

The UAV system operated at an altitude of 30 meters above ground, with the camera positioned in a nadir orientation for all data acquisitions, resulting in a ground sampling distance (GSD) of 20.8 mm·pixel^−^¹ for the images. Before each mission, the flight speed was configured to 7 km·h^−^¹, and flight routes were planned to ensure a minimum forward overlap of 70% and side overlap of 65% for all images, using the Autopilot flight control application (Hangar Technology, Austin, TX, USA).

### 2.3 Image processing

The multispectral images were processed using a pipeline developed in our previous study (Zhou et al., 2022), which included orthomosaic image generation, plot separation, and feature calculation. The multispectral images were processed using Pix4D Mapper (Pix4D, Lausanne, Switzerland) to generate orthomosaic images by importing all geo-referenced 5-band images and the CRPs for reflectance calibration (Zhou et al., 2019). The generated orthomosaic images were then processed using the Mapping Toolbox and Image Processing Toolbox of MATLAB (ver. 2019a, The MathWorks, Natick, MA, USA).

Individual plots (single-row for the PT, while four-row for the PYT and AYT) were delineated for each data collection by identifying the pixel positions in the orthomosaic images using corresponding GNSS coordinates. The latitude and longitude were extracted from the orthomosaic images using the *‘pixcenters’* function with a *‘makegrid’* option in the Mapping toolbox in MATLAB and were projected into the World Geodetic System 1984 (WGS84).

The GNSS positions of individual plots were obtained by manually separating the plots from one of the orthomosaic images in a trial. Rectangular masks were created to cover the full canopy of the individual plots. The GNSS positions were returned by applying the masks on the latitude and longitude matrices of the orthomosaic image. The pixel positions of the individual plots on the other images were obtained by matching the plot GNSS positions with the orthomosaic GNSS matrices.

The image background (soil, shadow, and plant residues) was removed from the separated images by detecting projected canopy contours using the ‘*activecontour*’ function (Whitaker, 1998) with the ‘Chan-Vese’ method (Chan and Vese, 2001). Pixels within a full contour were considered as foreground (soybean plants) while those outside contours were background (soil and residues). Contours with extreme small regions were identified as noises using the ‘*regionprops*’ function and then removed from the foreground. Seven image features (five VIs and two features from color space conversion) were calculated to predict the relative maturity date (RMD) of each soybean plot (**Table 1**). The seven image features were selected from a pool of 130 VIs and spectral band statistics evaluated in our previous work (Zhou et al., 2019). The feature selection procedure described in Zhou et al. (2019) was conducted to eliminate any negative effects of multicollinearity on model performance. It is worth noting that although field scouts determine physiological maturity primarily by pod color, here we used whole-canopy spectral and color features as a proxy for maturity stage. Previous studies have shown that time series of aerial images of late-season canopy senescence closely tracks pod maturation in soybean (Trevisan et al., 2020; Moeinizade et al., 2022; Pérez et al., 2024).

**Table 1:** Vegetation indices (VIs) used for predicting the soybean maturity date.

Name	Description	Formula
CCCI	Canopy Chlorophyll Content Index (El-Shikha et al., 2008)	$\frac{\frac{nir-re}{nir+re}}{NDVI}$
MTVI2	Modified Triangular VI (Hunt Jr. et al., 2011)	$\frac{1.5\times (1.2\times (nir-green)-2.5\times (red-green))}{\sqrt{{\left(3-nir\right)}^{2}-6\times nir+5\times \sqrt{red}-0.5}}$
BNDVI	Blue-normalized difference VI (Wang et al., 2007)	$\frac{nir-blue}{nir+blue}$
GLI	Green leaf index (Gobron et al., 2000)	$\frac{2\times green-red-blue}{2\times green+red+blue}$
CI	Coloration Index (Reis-Pereira et al., 2023)	$\frac{red-blue}{red}$
H	Hue (Cheng et al., 2001)	$\tan^{-1}(\frac{2\times red-green-blue}{30.5}\times (green-blue))$
V	Value (Cheng et al., 2001)	*rgb2hsv*

nir, near-infrared; re, red-edge; NDVI, Normalized Difference Vegetation Index; hsv, hue-saturation-value color space.

### 2.4 Maturity date and relative maturity date

**Figure 1:**
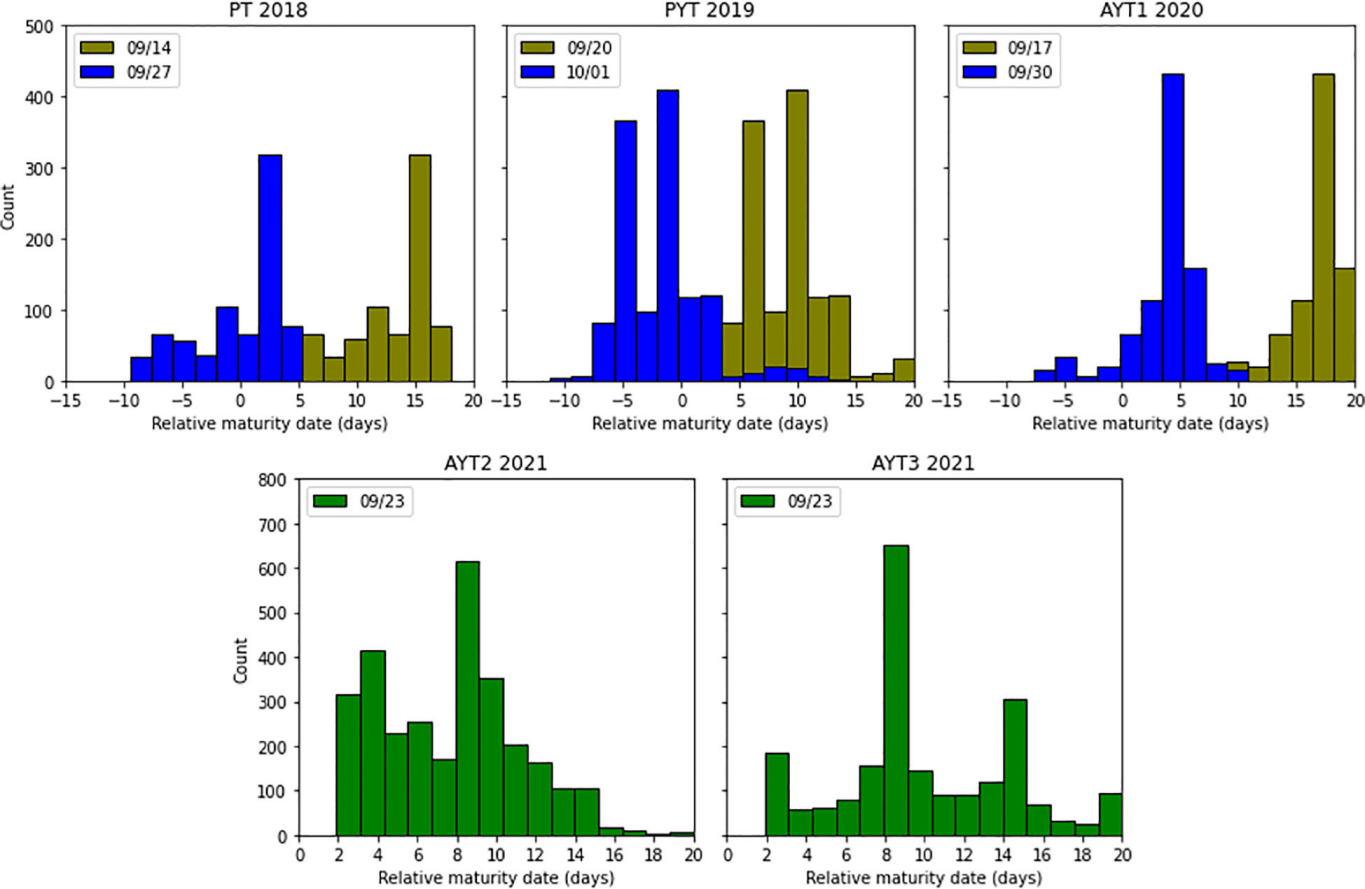
Distribution of relative maturity dates (RMDs) across the five trials. Bar colors indicate the timing of aerial image collection: blue and mustard-yellow denote the first and second collections in 2018–2020, respectively, while green represents the single collection in 2021.

The maturity date of each soybean plot was visually determined and recorded as the number of days after September 1 (day 1) when 95% of the pods in the two center rows of each plot achieved mature pod color. The RMD of a soybean plot was the number of days to its R8 date from the day of imaging and was calculated as the difference between the maturity date of each plot and each imaging date. Negative RMDs represent that the lines will mature after the imaging day, while positive values mean that the lines matured before that day. For example, if a soybean plot in PT matured at day 20 (i.e., September 20, 2018), its RMD to the imaging date of September 14 is -6 days. The RMD distributions of the three trials in 2018–2020 and two case study experiments in 2020 are shown in **Figure 1**.

### 2.5 Transfer learning model development

#### 2.5.1 Model architecture

**Figure 2:**
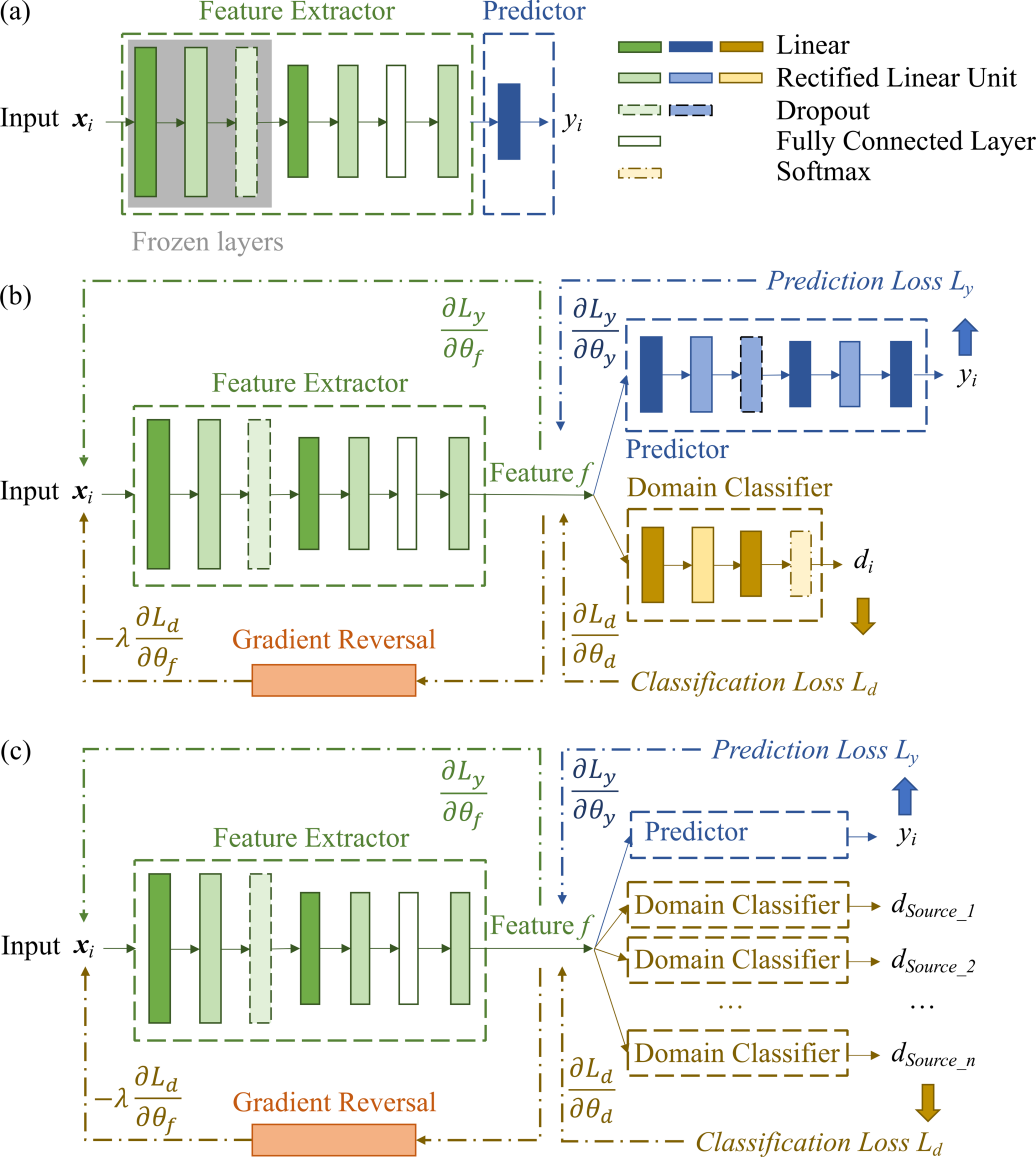
Architectures of the transfer learning models. **(a)** pre-training and fine-tuning, where parameters of the frozen layers were not updated during the fine-tuning, and those in the rest layers were tuned to fit the training set from the target domain; **(b)** single-source Domain Adaptation (DA) architecture; and **(c)** multiple-source DA architecture. When more than one source domain was considered in the source dataset, the multiple-source DA requires a domain classifier module for each source domain.

Three transfer learning techniques (**Figure 2**), namely pre-training and fine-tuning (Iman et al., 2023), single-source DA inspired by Ganin and Lempitsky (2015), and multiple-source DA inspired by Zhao et al. (2018), were applied and evaluated in this study. The models took ***x_i_*** as inputs (i.e., vegetation indices derived from UAV imagery) and *y_i_* as outputs (RMD predictions). During the model training stage, we defined the datasets with visually measured RMD from previous years as the source domain, and datasets from future years (i.e., assuming without visually measured RMD) as the target domain. Samples inherited domain labels *d_i_* ∈[0, 1] from this definition, indicating whether they belong to either the source (*d_i_* = 0) or target domain (*d_i_* = 1). The goal in model development is to output accurate RMD predictions for samples in the target domain leveraging the models mainly trained by data from the source domain.

Pre-training and fine-tuning. The architecture of the deep learning model for the pre-training and fine-tuning technique is shown in **Figure 2a**. It has a feature extractor that is a series of feed-forward layers mapping inputs to a feature vector. The predictor is a linear regression layer that converts the feature vector to an output *y_i_*. The mean squared error (MSE) between the model outputs and measured RMD was calculated as the prediction loss. During the pre-training, the model was trained from scratch with randomly initialized weights and learned the features and representations directly from the source dataset. Next, the pre-trained model was fine-tuned on a small amount of the target dataset (soybean checks), employing the pre-trained weights as a starting point. The earlier three layers of the model are frozen to preserve the learned representations from the source dataset, while the following layers were adjusted to the specific features in the target dataset.

Single-source DA. The architecture of the single-source DA model (**Figure 2b**) consists of a deep feature extractor, a predictor, and a domain classifier. The feature extractor maps inputs to a feature vector. Parameters of all the layers are denoted as *θ_f_*. The feature vector is then mapped by the predictor to generate outputs *y_i_*. Parameters in this module are denoted as *θ_y_*. Prediction loss *L_y_* is calculated using the MSE loss function to reflect the difference between the model outputs and the true RMD. At the same time, the same feature vector is mapped by the domain classifier to generate the domain label *d_i_*, indicating whether the samples belong to the source or target domain. Parameters in the domain classifier are denoted as *θ_d_*. Classification loss *L_d_* are calculated using the Negative Log Likelihood Loss (NLLL) function to reflect whether the distribution of the input features from the source and target domains are distinguishable.

In addition to minimizing the loss of the label predictor, the training objective also includes optimizing parameters *θ_f_* of the feature extractors *to maximize* the loss of the domain classifier (by making the feature distributions of the two domains as similar as possible), while simultaneously seeking the parameters *θ_d_* of the domain classifier that *minimize* the loss of the domain classifier. This is achieved by connecting the classification loss to the feature extractor through a gradient reversal layer that multiplies the gradient (of classification loss) by a certain negative constant. Thus during the backpropagation process, parameters *θ_f_* of the feature extractor are updated to minimize the label prediction loss, while maximizing the domain classification loss (Ganin and Lempitsky, 2015).

Multiple-source DA. Similar to the single-source DA model, the multiple-source DA model (**Figure 2c**) has three modules each consisting of a feature extractor, a predictor, and a domain classifier, guided by the core idea of learning input features that are indistinguishable between the multiple source domains and the target domain and representative of the RMD. If the training data are from multiple source domains (e.g., datasets from 2018 and 2019 in this study), the multiple-source DA allows multiple domain classifiers each mapping the feature vector to generate the label for individual domains (*d_i_source_j_*). Classification loss is also calculated using the NLLL function, taking all the domain labels into consideration. During the backpropagation, the loss contributes to tuning the parameters in both the feature extractor and all the domain classifiers (Zhao et al., 2018).

In this study, the three techniques employ a common feature extractor composed of a series of feed-forward linear layers. The differences between them were after the final layer of the extractor: in the fine-tuning approach, the extracted features were passed directly to an additional linear layer for prediction, whereas in the DA methods, these features were routed to both the predictor (another sequence of linear layers) and one or more domain classifiers. The architecture of the feature extractor can be modified as needed for other studies or applications.

#### 2.5.2 Datasets and model validation and testing

Generalizability of the three transfer-learning techniques was evaluated using data collected in five trials from 2018 to 2021. The models were developed with PyTorch (ver. 2.0.1) (Paszke et al., 2019) in Python (ver. 3.11.4, Python Software Foundation).

Model validation. Given that only two UAV flights were conducted around maturity in 2018-2020, lines maturing far earlier or later could not be reliably captured within the available imagery. Thus, samples with RMD values ≤-15 or ≥20 were excluded from the datasets, resulting in 1,514, 2,516, and 1,728 samples for the 2018, 2019, and 2020 datasets, respectively. For the pre-training and fine-tuning technique, 80% of the datasets in 2018 and 2019 were randomly split and combined to pre-train the model (source-training). The remaining 20% were combined to provide a baseline of the model performance before and after fine-tuning (source-testing). In 2020, 80% of the dataset were used for fine-tuning the models (target-training), while the remaining 20% were used to validate the fine-tuning performance (target-testing). For the DA models, 80% of the 2018 and 2019 datasets were used as source domains for training (source-training), and 80% of the 2020 datasets were used as the target domain for training (target-training). The remaining 20% in each of the source and target domains were used as testing datasets to validate the generalizability (source-testing and target-testing). The model performance was evaluated using the *R^2^* (calculated with the ‘*r2_score*’ function in Python ‘*scikit-learn*’ library) and the *RMSE* between the predicted and visual RMD.

Model testing on independent environments. To evaluate model generalizability and transfer learning performance, we trained the three models using datasets from 2018 to 2020 and tested them on the independent 2021 trials. Samples with RMD values ≤-15 or ≥ 20 were excluded to ensure alignment with imaging dates, resulting in 2,967 samples for AYT2 and 2,160 for AYT3. Datasets were split to simulate practical fine-tuning and application. For the pre-training and fine-tuning technique, 80% of the 2018–2020 data were allocated for training and 20% for validation. In 2021, check lines (395 samples for AYT2 and 120 for AYT3) served for fine-tuning, and the breeding lines (2572 samples for AYT2 and 2040 for AYT3) were used for testing the model. For the DA models, all 2018–2020 data were used for training, with 2021 data used for both training and testing.

## 3 Results

### 3.1 Performance of pre-training and fine-tuning

**Figure 3:**
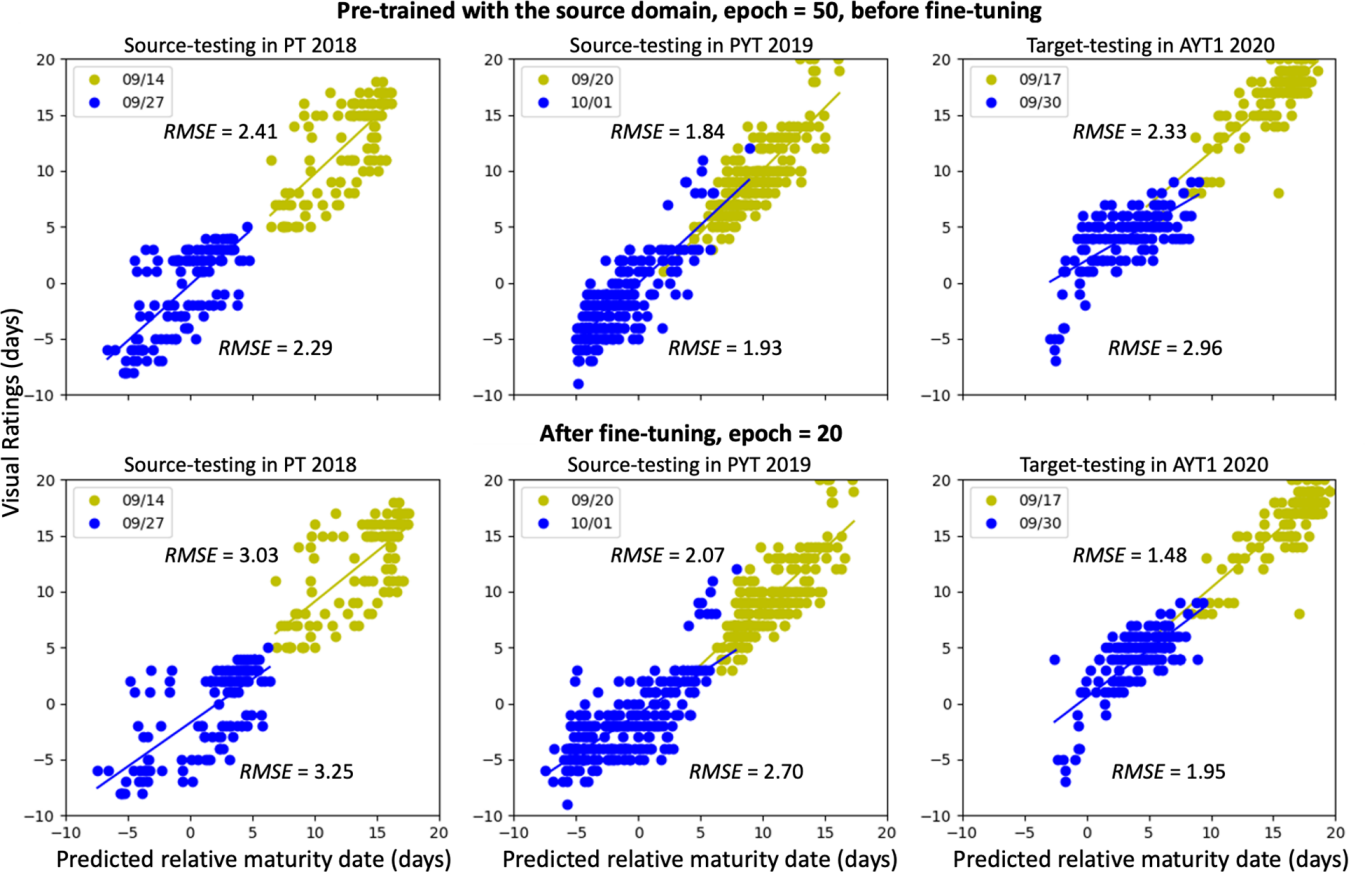
Performance of pre-training and fine-tuning. Dot colors in each plot indicate data from two data collections. Once in mid-September (in yellow) and once at the end of September (blue) for all three years.

The pre-trained model (**Figure 2a**), which was trained with combined data from 2018 and 2019 (source domain) and fine-tuned on 2020 data (target domain), shows distinct performance patterns when was evaluated on the testing datasets from both domains before and after fine-tuning (**Figure 3**). Before fine-tuning and considering imagery collected from two drone flights, the models achieved *RMSE* values within 2.29-2.41 days (2018) and 1.84-1.93 days (2019) on source-domain test data, with the higher error in 2018 attributable to greater phenotypic diversity and fewer samples in the progeny trial. On the unseen 2020 target domain, pre-fine-tuning *RMSE* was within 2.33-2.96 days. After fine-tuning for 20 epochs on the 2020 training set, target-domain performance improved substantially, reaching an *RMSE* within 1.48-1.95 days, representing a 34-36% reduction in error compared to the pre-fine-tuning baseline.

For consistent comparison with DA results, target-domain performance in **Figure 3** was obtained by fine-tuning using 80% of the 2020 dataset. To systematically assess the impact of fine-tuning data volume, **Table 2** reports model performance before and after fine-tuning with 10%–90% of the target-domain training data. The *R²* and *RMSE* values were calculated combining data from two flight dates. They were reported to compare model performance across training scenarios rather than indicating predictive accuracy. Post-fine-tuning *RMSE* ranged from 1.71 days (10% data) to 1.51 days (70% data), with only minor variations across ratios and no clear monotonic trend. This indicates that substantial performance gains are achievable even with limited target-domain samples (as low as 10%) and confirms effective knowledge transfer from pre-trained models on historical data to new environments using minimal additional labeled data.

**Table 2:** Model simulation performance before and after fine-tuning with different ratios of the target domain dataset.

Fine-tuning ratio	Before fine-tuning		After fine-tuning					
	2020		2018		2019		2020	
	*R^2^*	*RMSE*	*R^2^*	*RMSE*	*R^2^*	*RMSE*	*R^2^*	*RMSE*
0.1	0.87	2.40	0.77	3.20	0.86	2.23	0.94	1.71
0.2	0.87	2.41	0.79	3.13	0.86	2.33	0.94	1.69
0.3	0.88	2.38	0.78	3.28	0.85	2.50	0.94	1.61
0.4	0.88	2.38	0.78	3.31	0.85	2.56	0.94	1.63
0.5	0.87	2.40	0.78	3.36	0.85	2.51	0.94	1.59
0.6	0.87	2.39	0.78	3.34	0.86	2.43	0.95	1.56
0.7	0.87	2.37	0.78	3.31	0.86	2.45	0.95	1.51
0.8	0.87	2.41	0.80	3.25	0.85	2.54	0.95	1.54
0.9	0.86	2.47	0.81	3.22	0.86	2.45	0.95	1.57

Results show model performance on the remaining proportion of the target domain data.

The *R²* and *RMSE* values were calculated combining data from two flights and only used to compare performance across training scenarios rather than indicating predictive accuracy. The model was pre-trained for 50 epochs and fine-tuned for another 50 epochs. Performance for 2018 and 2019 before fine-tuning is shown in **Figure 3**.

**Figure 4:**
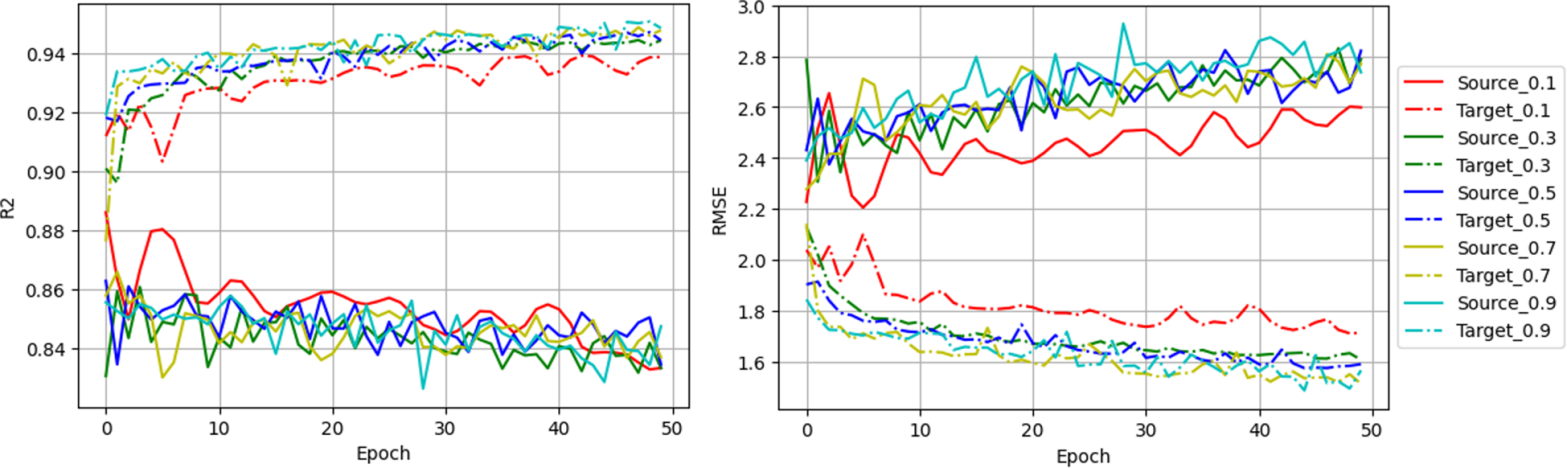
Model simulation performance along epochs during the fine-tuning. Solid and dash-dot lines indicate source testing accuracy and target testing accuracy, respectively. Line colors indicate the models fine-tuned with different ratios of the target domain. The *R²* and *RMSE* values were calculated combining data from two flights and only used to compare performance across training scenarios rather than indicating predictive accuracy.

**Figure 4** shows the trend of *R^2^* and *RMSE* during the fine-tuning. In target domain, *R^2^* increased shapely and *RMSE* dropped quickly within the first few epochs, stabilizing after 20 epochs regardless of the data ratio used. Target domain consistently outperformed source domain, suggesting effective generalization after fine-tuning. In contrast, source domain metrics fluctuated strongly and showed a slight overall decline as epochs increased, which was an expected consequence of progressive adaptation to the target domain. Lower fine-tuning ratios led to more pronounced early fluctuations in the source domain but greater stability in later epochs, however notably, the 10% ratio yielded the poorest final *R²* and highest *RMSE*. Balancing performance across both domains, a suitable number of epochs in fine-tuning plays an important role in reaching a balanced performance. In our case, we considered 20 epochs the optimal stopping point.

### 3.2 Performance of single-source domain adaptation

**Figure 5:**
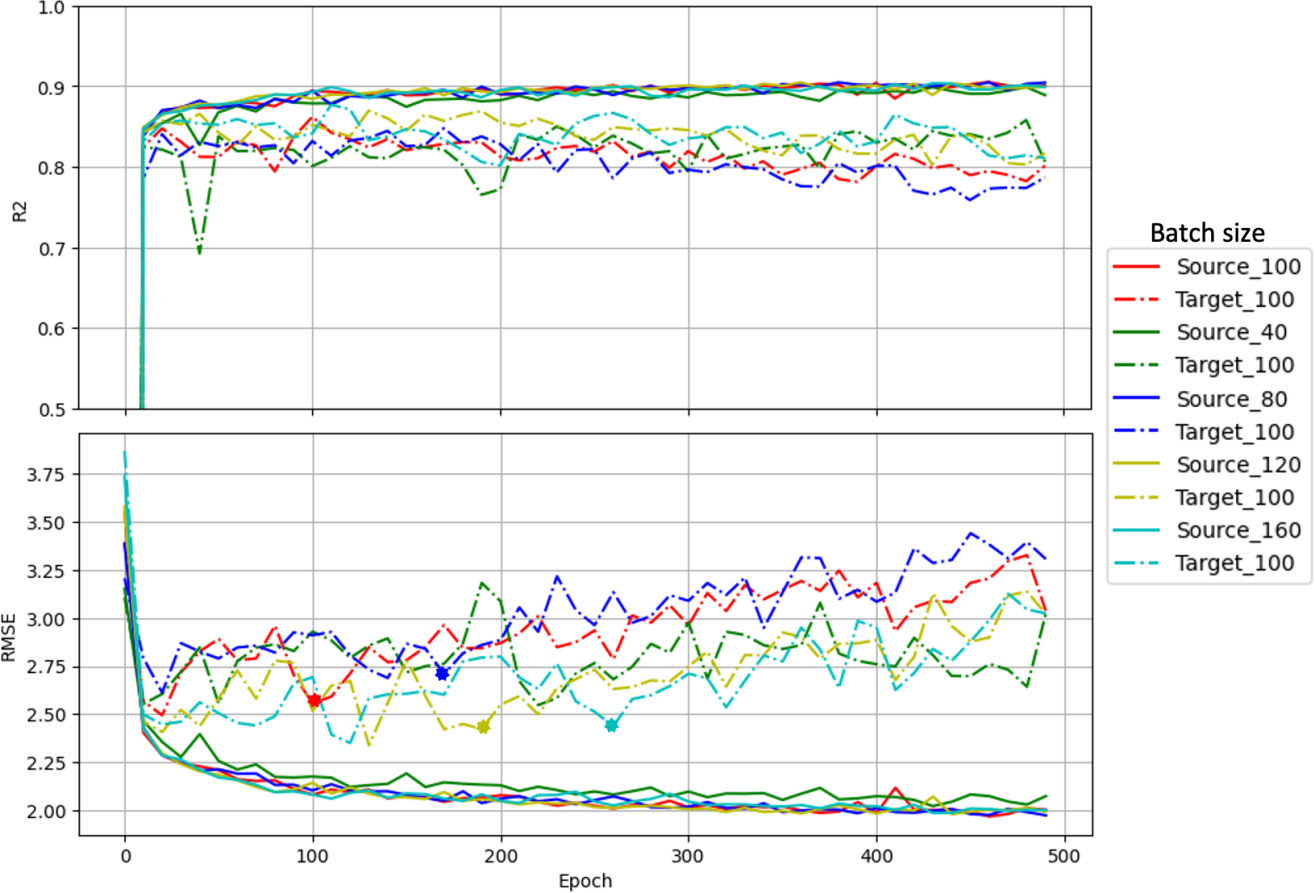
Model simulation performance of the single-source domain adaptation across epochs. Solid and dash-dot lines indicate source testing accuracy and target testing accuracy, respectively. Line colors indicate models trained with different batch sizes of the source domain. Each point value is the average of the following 10 points. The stars indicate the turning point (if applicable) where the overall target testing accuracy started decreasing. The *R²* and *RMSE* values were calculated combining data from two flights and only used to compare performance across training scenarios rather than indicating predictive accuracy.

**Figure 5** shows the testing performance of the single-source DA model across training epochs and batch-size configurations. As training progressed, source-domain accuracy steadily improved while target-domain accuracy gradually declined, reflecting the model’s increasing fit to labeled source data. This pattern highlights the importance of early stopping to preserve target-domain generalization, similar to observations in fine-tuning.

**Figure 6:**
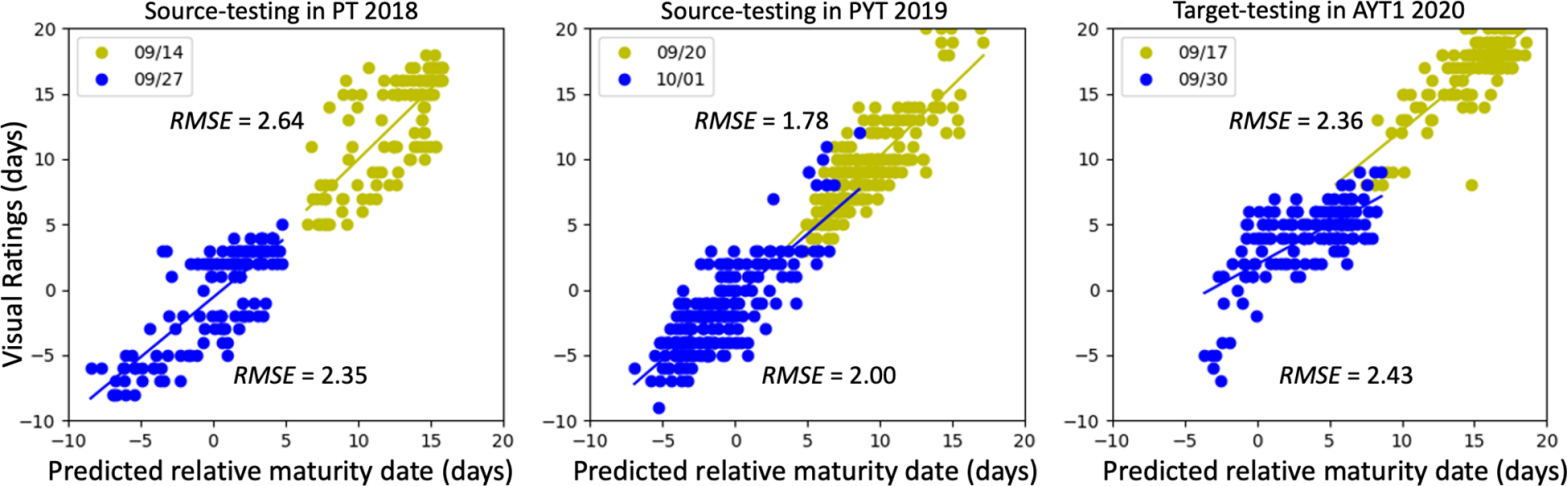
Performance of single-source domain adaptation. Dot colors indicate data from two data collections. Once in mid-September (in yellow) and once at the end of September (blue) for all three years. The model was trained for 100 epochs with a batch size of 100.

In addition, batch size controlled the relative contribution of source and target samples per epoch (target batch size fixed at 100, yielding 14 batches). As source batch sizes increased from 40 to 200, there were fewer, equal, or more source samples per epoch compared with the target domain. Target-domain performance exhibited pronounced epoch-to-epoch fluctuations that were, however, insensitive to source batch size, whereas source-domain performance remained stable. Optimal balance occurred when the source batch size was set at 100, yielding *RMSE* within 2.10-2.64 days (2018 source), 1.78-2.00 days (2019 source), and 2.36-2.43 days (2020 target), as shown in **Figure 6**. This is also the turning point where the target testing accuracies kept decreasing. However, there was no clear turning point within the 500 epochs for the batch sizes of 40 and 60. The turning point for batch sizes of 80 and 120 were observed at around 200 and the one for batch sizes of 140 to 200 was at around 250. Thus, the results indicate that greater source–target sample imbalance delays convergence but does not substantially alter peak target-domain performance, highlighting the robustness of the adaptation strategy even under highly asymmetric data regimes.

### 3.3 Performance of multiple-source domain adaptation

**Figure 7:**
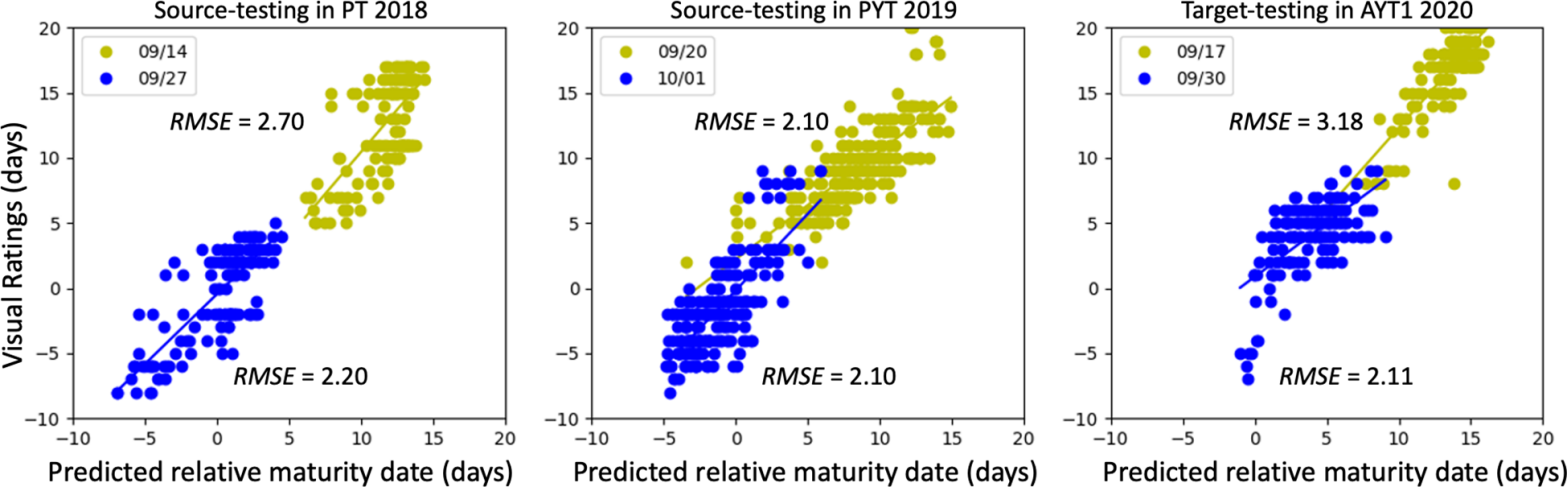
Performance of multiple-source domain adaptation. Dot colors indicate data from two data collections. Once in mid-September (in yellow) and once at the end of September (blue) for all three years. The model was trained for 100 epochs with a batch size of 100.

**Figure 7** shows the performance of the multiple-source DA model. The best results considering imagery collected from two drone flights, were obtained using a batch size of 100 per domain after 80 training epochs, yielding *RMSE* values within 2.20-2.70 days (2018 source), 2.10 days (2019 source), and 2.11-3.18 days on the 2020 target domain. Compared with the single-source DA model, the multiple-source approach consistently underperformed on both source and target domains, which likely stems from two reasons. First, treating the 2018 and 2019 datasets as separate source domains violates a core assumption of domain adaptation, i.e., source and target distributions should be similar (Ganin and Lempitsky, 2015). In the single-source model, combining 2018 and 2019 data creates a broader, more comprehensive, and representative source distribution that better aligns with the target domain. In contrast, splitting the sources introduces distributional mismatches, making effective alignment more difficult. Second, the increased model complexity required to simultaneously minimize multiple domain losses (one for each source) and the task-specific prediction loss appears to hinder optimization. The multiple-source model must reconcile potentially conflicting gradients across domains, whereas the single-source model focuses on a single, unified adaptation objective. Consequently, the multiple-source configuration struggled to achieve the same level of feature alignment and predictive accuracy as the single-source model.

### 3.4 Model performance between two image collections

Across the two flights in each year, model performance varied noticeably by transfer learning techniques but slightly by imaging timing. The pre-training and fine-tuning model consistently delivered the highest accuracy on the 2020 target dataset, achieving *RMSE* = 1.47 days (*R²* = 0.72) on the first imaging date and *RMSE* = 1.71 days (*R²* = 0.61) on the second. The relatively small performance gap between dates confirms that imaging timing is flexible for this approach. Satisfactory predictions can be obtained throughout the mid- to late-September window (107–120 DAP) without strict dependence on capturing peak senescence. In contrast, both DA methods exhibited greater sensitivity to imaging date. Single-source DA performed slightly better on the earlier imaging date while multiple-source DA performed remarkably better on the later date.

### 3.5 Model performance on two independent trials

**Figure 8:**
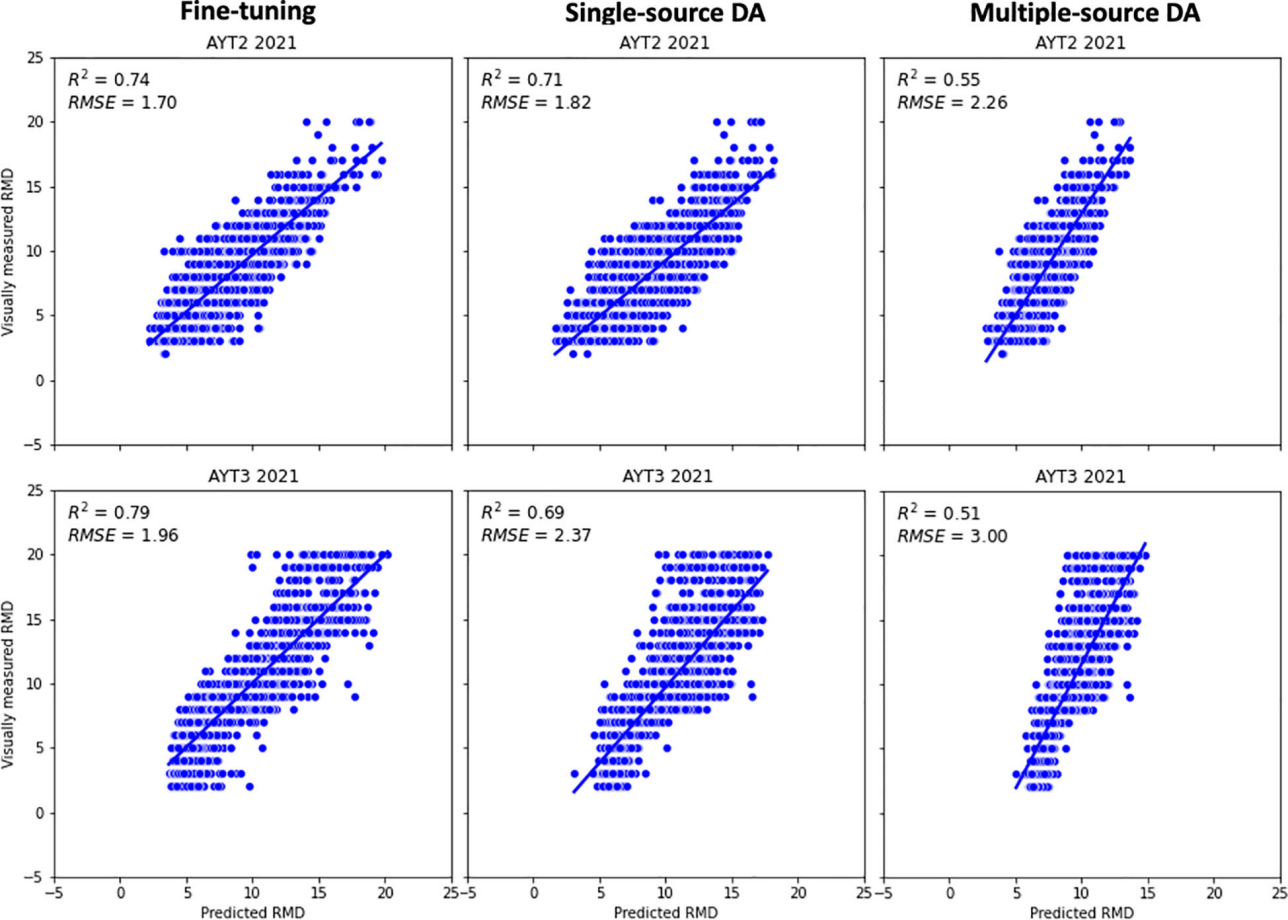
Performance of three transfer learning techniques, fine-tuning, single-source domain adaptation (DA), and multiple-source DA, using datasets from 2018 to 2020 as source domain(s) and two 2021 trials as target domains (AYT2 and AYT3).

To further assess the model transferability, the three models were trained on 2018–2020 data (source domains) and evaluated on two completely unseen 2021 fields (target domains), as shown in **Figure 8**. The pre-trained model on source domains was fine-tuned with only check lines in each trial (395 lines in AYT2 and 120 lines in AYT3). Among the three transfer learning techniques, fine-tuning achieved the best performance, with *RMSE* of 1.70 days (AYT2) and 1.96 days (AYT3) across all non-check breeding lines. Before fine-tuning with the check lines, the model yielded *RMSEs* of 2.20 and 2.60 days for AYT2 and AYT3, respectively.

The DA models used all samples from the two new trials for training and testing to simulate practical unsupervised deployment. It should be noted that target domain labels (i.e., the visual ratings of RMD) were not used during training and thus all its samples remained unseen by the models. For both AYT2 and AYT3, single-source DA with *RMSEs* of 1.82 and 2.37 days outperformed multiple-source DA (*RMSE* = 2.26 and 3.00 days, respectively).

## 4 Discussion

This study demonstrates the efficacy of transfer learning techniques in enhancing model performance and generalizability in predicting soybean RMD using UAV imagery. By comparing fine-tuning, single-source DA, and multiple-source DA on a multi-year dataset, we provide actionable insights into their practical utility for soybean breeding programs.

Pre-training with fine-tuning delivered the highest predictive accuracy, achieving *RMSE* within 1.47-1.71 days when transferring from 2018–2019 to 2020 and within 1.70-1.96 days when transferring from 2019–2020 to two completely independent 2021 fields. The errors fall within the two-day threshold considered acceptable by breeders for maturity-group assignment (Pérez, 2025; Zhou et al., 2019). Notably, several prior studies reported slightly lower errors, for example, *RMSE* of 1.3 to 1.8 days by Pérez et al. (2024) on a three-year (2018-2020) UAV imagery dataset. However, the low errors resulted from the cross-validation process that included subgroups of data from all environments. In their study, when the model was trained with data from 2018–2019 and check lines from 2020, the prediction errors increased to about 2.1 days on remaining the 2020 data. In addition, Moeinizade et al. (2022) reported mean average errors of 0.9 to 2.5 days for predicting maturity date for six trials. However, the model was exposed to data from all environments and not evaluated on completely independent data. In Trevisan et al. (2020), data from five independent trials were collected, and the models trained with data from single trials were tested in all other trials. The overall performance of the models trained and evaluated within the same trial indicated *RMSE* values lower than 2 days; however, the performance of models trained in other trials varied, ranging from 1.4 to 5.5 days.

The previous study in Pérez et al. (2024) confirmed that incorporating check plots from an independent test year into training led to reduced prediction bias, improving model generalizability on independent datasets. Transfer learning techniques enable the seamless incorporation of these data and update the model without full retraining, unlike what happens when using traditional machine learning models. In soybean breeding trials, a small number of check samples are always planted with the new breeding materials as a reference to determine relative maturity groups. Maturity dates of check samples will always be visually assessed. This small, labeled set could be leveraged for fine-tuning pre-trained models on historical trials for new experiments. The low sensitivity of the fine-tuning performance to data volume (as shown when the *RMSE* values varied only from 1.71 to 1.51 days across 10–90% ratios in **Table 2**), offers a reference for practical use of this approach. Additionally, from the model performance throughout the change of epochs (**Figure 4**), we observed that although fine-tuning with more epochs decreased the target errors, it significantly increased the source errors. A suitable number of epochs in the fine-tuning plays an important role in reaching a balanced performance. In this case, we found that fine-tuning for 20 epochs reached the sweet spot for the balance.

Domain adaptation techniques offered fully unsupervised alternatives but proved more sensitive to distributional alignment, as expected from theory (Ganin and Lempitsky, 2015; Peng et al., 2019). The single-source DA consistently provided in both AYT2 and AYT3 better generalization than multiple-source DA, which showed context-dependent variability (**Figure 8**). This variability could be interpreted as suggested by Trevisan et al. (2020) that large residuals in trials with varied leaf and pod senescence patterns would be influenced by environmental factors and their interaction with genotype. In addition, in multiple-source DA, inconsistent systematic biases (structural errors) across individual source domains can be amplified if the model overly aligns the target domain to one biased source while ignoring others (Peng et al., 2019; Zhao et al., 2018). In our study, multiple-domain DA occasionally outperformed single-source DA on specific imaging dates (e.g., *RMSE* = 2.11 days versus 2.43 days on the target domain on September 30, 2020), although it performed worse in most scenarios (**Table 3**). This demonstrated the amplification of structural bias from the “favored” source domain, as this better performance remains above the threshold of 2 days. This led us to conclude that in predicting maturity date, single-source DA is recommended over multiple-source DA for practical deployment unless a much larger and more diverse historical archive is available.

**Table 3:** Model performance of two imaging dates.

Transfer learning methods	Years											
	2018 (source)				2019 (source)				2020 (target)			
	09/14 (108)		09/27 (121)		09/20 (109)		10/01 (120)		09/17 (107)		09/30 (120)	
	*R^2^*	*RMSE*	*R^2^*	*RMSE*	*R^2^*	*RMSE*	*R^2^*	*RMSE*	*R^2^*	*RMSE*	*R^2^*	*RMSE*
Pre-training and fine-tuning	0.42	3.03	0.23	3.25	0.57	2.07	0.35	2.70	0.72	1.47	0.61	1.71
Single-source DA	0.55	2.64	0.58	2.35	0.68	1.78	0.63	2.00	0.30	2.36	0.35	2.43
Multiple-source DA	0.46	2.70	0.31	2.20	0.57	2.10	0.62	2.10	0.30	3.18	0.41	2.11

Numbers in parentheses show the days after planting (DAP) in the year. Discrepancies between results in this table and **Figure 3** arose from different groups of data used in fine-tuning and testing. In **Figure 3**, the model was fine-tuned with 80% of the 2020 data and tested on the remaining, whereas in this table, the model was fine-tuned on check lines and tested on other lines.

Imaging timing further highlighted the specific tolerance of the methods. The pre-training and fine-tuning method tolerated a wide phenological window (107–120 DAP), with both dates yielding the highest accuracies among the evaluated methods. It suggests that it is the most practical method for predicting maturity date on new trials. Optimal imaging timing for the DA appeared unpredictable, as the performance mainly depend on the alignment between target and source distributions rather than absolute developmental stage.

In this study, the seven image features were chosen from a large set of 130 image features described in our previous work (Zhou et al., 2019). Selection criteria combined a strong individual linear correlation with visual ratings of maturity date and minimal multicollinearity with other features. These features are particularly well-suited for soybean maturity prediction because they collectively track the sequential physiological, structural, and visual changes that occur during the critical late-reproductive stages. Canopy Chlorophyll Content Index integrates NDVI and Normalized Difference Red Edge (NDRE) to provide a sensitive indicator of canopy nitrogen status (Cammarano et al., 2011), which declines progressively as pods fill and leaves senesce (Craft et al., 2019). Modified Triangular Vegetation Index and GLI respond to combined changes in chlorophyll concentration, leaf area, and canopy architecture (Hunt Jr. et al., 2011; Gobron et al., 2000). They effectively capture the progressive loss of green canopy density and the exposure of yellowing foliage that characterize the transition from active seed fill to full maturity (Xing et al., 2020). Blue-Normalized Difference Vegetation Index and CI emphasize reflectance changes in the blue region, which strengthen the detection of the pronounced shift from green to yellow/brown pigments (Kumar et al., 2023). Hue and value from the HSV (hue-saturation-value) color space directly encode the perceptual changes. Hue shifts from green (~120°) toward yellow (~60°), while Value decreases as the canopy dries and darkens. These simple yet intuitive metrics quantify crop changes during the transition from immaturity to maturity.

Despite these discoveries, the study relied on only three historical years as source domains, limiting the data availability for incorporating varying patterns between images and maturity data under various genotype-by-environment effects. Additionally, while check-based fine-tuning proved highly effective, its success could depend on accurate visual scoring of those checks and variability between individuals who take the notes. Future work should prioritize expanding source-domain archives across more seasons and locations to strengthen unsupervised DA. Exploring foundation models pre-trained on large, diverse imagery datasets may ultimately bridge the remaining performance gap between supervised fine-tuning and fully unsupervised adaptation.

## 5 Conclusion

This study demonstrates the potential of using transfer learning in enhancing the generalization of deep learning models for predicting soybean RMD across years and locations, providing a solution to overcome a major barrier in high-throughput phenotyping in breeding programs. Pre-training on historical datasets followed by light fine-tuning using only the small set of visually scored check cultivars that are routinely planted in every soybean breeding trial emerged as the most effective and practical strategy. It achieved prediction errors within two days on completely independent trials while requiring the labeling of only 10% of the total new data for fine-tuning. Precise imaging date is proved not required with flexibility within the late-reproductive window (107–120 DAP), meaning that this approach transforms a traditionally labor-intensive trait into one that can be monitored at scale with minimal human input beyond standard check-plot assessments.

Unsupervised DA techniques, although eliminating any need for target-domain labels, exhibited greater sensitivity to distributional alignment between source and target environments. Single-source DA, by pooling all prior data into a single, broader source distribution, provided more stable and predictable generalization than multiple-source DA, which occasionally outperformed when the target environment resembled one specific historical year but otherwise underperformed. These findings reinforce the theoretical expectation that successful unsupervised adaptation hinges on comprehensive source-domain coverage of the variability.

Collectively, the results establish a clear, actionable pathway for soybean breeding programs: maintain an evolving pre-trained model on all past UAV campaigns and, each new season, fine-tune it using the handful of check cultivars already present in every trial. This hybrid strategy achieves the accuracy of fully supervised approaches while minimizing the labeling efforts. By enabling reliable RMD prediction in unseen environments with almost no added phenotyping cost, transfer learning paves the way for faster and more precise maturity-group assignment across broader geographic regions.

## Data Availability

The raw data supporting the conclusions of this article will be made available by the authors, without undue reservation.
